# Impact of Body Composition on the Risk of Hepatocellular Carcinoma Recurrence After Liver Transplantation

**DOI:** 10.3390/jcm8101672

**Published:** 2019-10-13

**Authors:** Karolina Grąt, Ryszard Pacho, Michał Grąt, Marek Krawczyk, Krzysztof Zieniewicz, Olgierd Rowiński

**Affiliations:** 1 Second Department of Clinical Radiology, Medical University of Warsaw, 02-097 Warsaw, Poland; 2 Department of General, Transplant and Liver Surgery, Medical University of Warsaw, 02-097 Warsaw, Poland,

**Keywords:** obesity, hepatocellular carcinoma, liver transplantation, subcutaneous fat, visceral fat, computed tomography, adipose tissue, sarcopenia

## Abstract

Background: Body composition parameters are reported to influence the risk of hepatocellular carcinoma (HCC) recurrence after liver resection, yet data on patients undergoing liver transplantation are scarce. The aim of this study was to evaluate the impact of the amount of abdominal adipose tissue and skeletal muscles on the risk of HCC recurrence after liver transplantation. Methods: This was a retrospective observational study performed on 77 HCC patients after liver transplantation. Subcutaneous fat area (SFA), visceral fat area, psoas muscle area and total skeletal muscle area were assessed on computed tomography on the level of L3 vertebra and divided by square meters of patient height. The primary outcome measure was five-year recurrence-free survival. Results: Recurrence-free survival in the entire cohort was 95.7%, 90.8%, and 86.5% after one, three, and five years post-transplantation, respectively. SFA was significantly associated with the risk of HCC recurrence (*p* = 0.013), whereas no significant effects were found for visceral fat and skeletal muscle indices. The optimal cut-off for SFA for prediction of recurrence was 71.5 cm^2^/m^2^. Patients with SFA < 71.5 cm^2^/m^2^ and ≥71.5 cm^2^/m^2^ exhibited five-year recurrence-free survival of 96.0% and 55.4%, respectively (*p* = 0.001). Conclusions: Excessive amount of subcutaneous adipose tissue is a risk factor for HCC recurrence after liver transplantation and may be considered in patient selection process.

## 1. Introduction

Liver transplantation remains the mainstay of treatment for patients with hepatocellular carcinoma (HCC) at low risk of post-transplant tumor recurrence [1]. For more than two decades, appropriate selection of HCC patients for transplantation was accomplished through strict utilization of the Milan criteria (one tumor < 5 cm or two to three tumors < 3 cm, no macrovascular invasion, no extrahepatic spread) [2]. Currently, however, there is a shift towards more precise selection of patients using a combination of morphological criteria with surrogates of tumor biological aggressiveness, such as serum alpha-fetoprotein concentration [3,4,5,6,7,8]. Despite these improvements, a remarkable proportion of patients beyond the Milan criteria at increased risk of HCC recurrence remains eligible for transplantation according to recent proposals [9,10]. Therefore, further refinement of patient selection strategy is warranted.

Obesity is a known factor predisposing to development of cancer [11]. Increased amount of adipose tissue is widely reported to be a negative prognostic factor in patients with various malignancies [12,13,14]. Aside from obesity, generalized loss of skeletal muscle mass (sarcopenia) is also predictive of negative outcomes in cancer patients [15,16,17,18,19]. The negative effects of both sarcopenia and accumulation of adipose tissue were confirmed in patients undergoing liver resections for HCC with respect to recurrence and survival, yet the results of available studies are inconsistent [20,21,22]. The potential mechanisms responsible for the influence of the quantity of adipose tissue and skeletal muscles comprise systemic and tumor-directed actions of various molecules, namely adipokines and myokines [23,24]. Increased levels of leptin and decreased levels of adiponectin are common in obesity and related to increased inflammation, angiogenesis, cell proliferation, and metastatic potential of cancer cells [25]. Decreased levels of interleukin-15 observed in sarcopenia contribute to immune senescence. Notably, altered function of natural killer cells was also found in case of obesity [24,26]. In contrast to several previous studies focused on the impact of body composition on the outcomes of HCC patients undergoing hepatectomy, data with respect to patients undergoing liver transplantation is scarce. Liver transplantation is associated with removal of the underlying liver disease along with potential intrahepatic micrometastases, occurrence of ischemia-reperfusion injury directly modifying the risk of recurrence [27], and immune suppression. Therefore, the findings from liver resection studies cannot be extrapolated. The aim of this study was to evaluate the impact of body adipose and muscle contents on the risk of HCC recurrence after liver transplantation.

## 2. Materials and Methods

**Figure 1:**
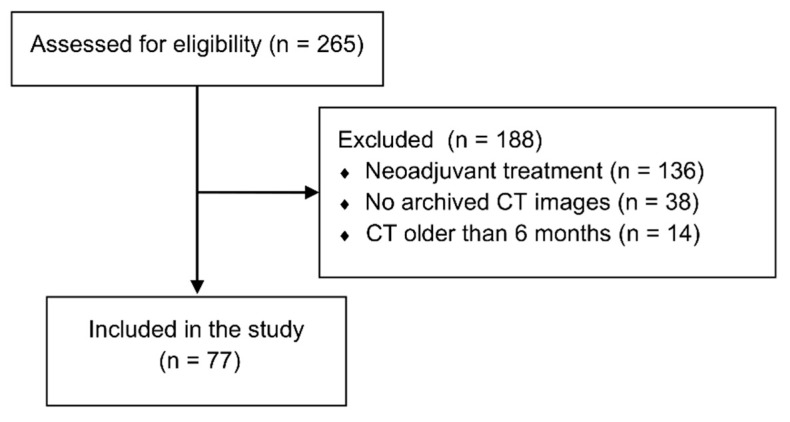
Study flowchart.

This retrospective observational study was performed on the data of 77 HCC patients after liver transplantations performed in the period between January 2006 and June 2017 in the Department of General, Transplant and Liver Surgery of the Medical University of Warsaw. The sole inclusion criterion was histologically confirmed HCC in the explanted liver. Exclusion criteria comprised a lack of available images from multiphasic computed tomography (CT) performed within 6 months preceding transplantation and utilization of neoadjuvant treatment (Figure 1). The study protocol was approved by the local ethics committee of the Medical University of Warsaw (AKBE/231/2017). Due to the retrospective nature, the need for informed consent was waived. 

**Figure 2:**
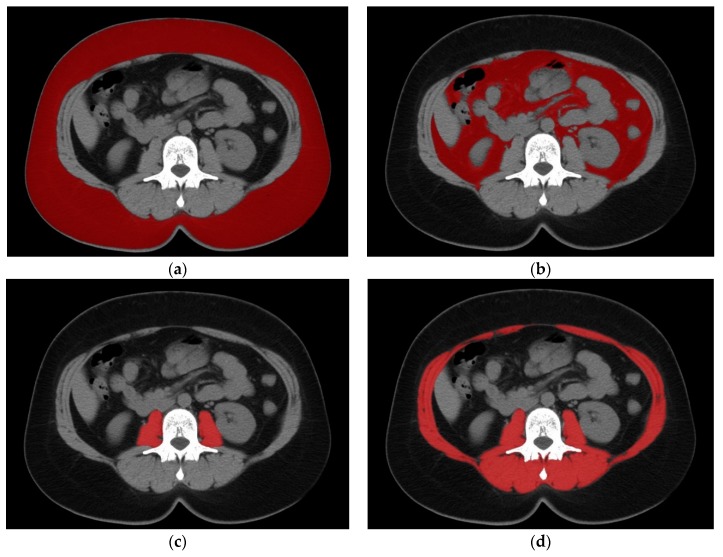
Examples of muscle and adipose tissue measurements: (**a**) Subcutaneous Fat Area (**b**) Visceral Fat Area (**c**) Psoas Major Area (**d**) Total Skeletal Muscle Area. (**a**) red zone represents Subcutaneous Fat Area, in this patient it was 332.6 cm^2^/m^2^ (**b**) red zone represents Visceral Fat Area, in this patient it was (138.9 cm^2^/m^2^) (**c**) red zone represents Psoas Major Area, in this patient it was 16.8 cm^2^/m^2^ (**d**) red zone represents Total Skeletal Muscle Area, in this patient it was 123.2 cm^2^/m^2^.

The primary outcome measure was 5-year recurrence-free survival, defined as time between liver transplantation and tumor recurrence. Observations were censored at last available follow-up, death for non-recurrence related causes, or 5 years post-transplantation, whichever occurred earlier. Details on surgical technique, perioperative care, immunosuppression protocol and post-transplant follow up were described elsewhere [28,29]. The primary factors of interest were subcutaneous (SFA), visceral (VFA), and total fat area (TFA), psoas muscle area (PMA), and total skeletal muscle area (TSMA) expressed in cm^2^ per 1 m^2^ of patient height. The areas of tissues of interest were calculated in Osirix software (Pixmeo, Switzerland) in a selected region of interest based on the range of Hounsfield units (HU). All calculations were performed using computed tomography scans on the level of the transverse processes of the third lumbar vertebra. The HU ranges were: −150 to −30 HU for VFA; −150 to −50 HU for SFA, and −30 to +150 HU for PMA and TSMA. TFA was defined as the sum of VFA and SFA. Examples of calculation of SFA, VFA, PMA, and TSMA are presented in Figure 2a–d, respectively. 

Quantitative variables were expressed as medians with interquartile ranges (IQRs) and qualitative variables were expressed as numbers with percentages. Chi^2^ test and Kruskall–Wallis test were used for intergroup comparisons of qualitative and quantitative variables, respectively. Spearman’s correlation coefficient was applied for analyses of correlations. Kaplan–Meier method was used for calculation of recurrence-free survival. Log-rank test was used for comparison of survival curves. Cox proportional hazards regression was used to evaluate risk factors for inferior recurrence-free survival. Receiver operating characteristics (ROC) analyses were performed to determine optimal cut-offs for selected body composition parameters in prediction of post-transplant HCC recurrence. Hazard ratios (HRs) and areas under the curve (AUCs) were presented with 95% confidence intervals (95% CI). Two-tailed *p* < 0.05 was considered to indicate statistical significance. All statistical analyses were performed using STATISTICA version 13 software (Dell Inc., Tulsa, USA). 

## 3. Results

Baseline characteristics of the 77 patients included in the study cohort are summarized in Table 1. Median VFA, SFA, and TFA were 27.8 cm^2^/m^2^ (IQR: 20.3–46.6), 51.7 cm^2^/m^2^ (IQR: 37.3–72.3), and 83.4 cm^2^/m^2^ (IQR: 58.7–110.9), respectively. Median Body Mass Index (BMI) was 25.2 kg/m^2^ with IQR of 23.3–28.3.

Body mass index was significantly correlated to VFA (*p* < 0.001), SFA (*p* < 0.001), TFA (*p* < 0.001), PMA (*p* < 0.001), and TSMA (*p* < 0.001) in all patients and separately in males and females (Table 2). None of the analyzed body composition parameters was significantly correlated with patient age or model for end-stage liver disease score.

Median follow-up period was 42 months. There were a total of seven patients who developed post-transplant HCC recurrence after a median of 12 months post-transplantation. Recurrence-free survival rates at one, three, and five years post-transplantation were 95.7%, 90.8%, and 86.5%, respectively.

Both SFA (*p* = 0.013) and TFA (*p* = 0.017) were significantly associated with the risk of post-transplant HCC recurrence. The risk of recurrence was not associated with VFA (*p* = 0.264), PMA (*p* = 0.141), and TSMA (*p* = 0.618). Out of the other factors, significant predictors of recurrence comprised diameter of the largest nodule (*p* = 0.005), alpha-fetoprotein concentration (*p* = 0.006), fulfillment of Milan criteria (*p* < 0.001), and presence of microvascular invasion (*p* = 0.022). The results of analyses of risk factors for tumor recurrence are summarized in Table 3.

**Figure 3:**
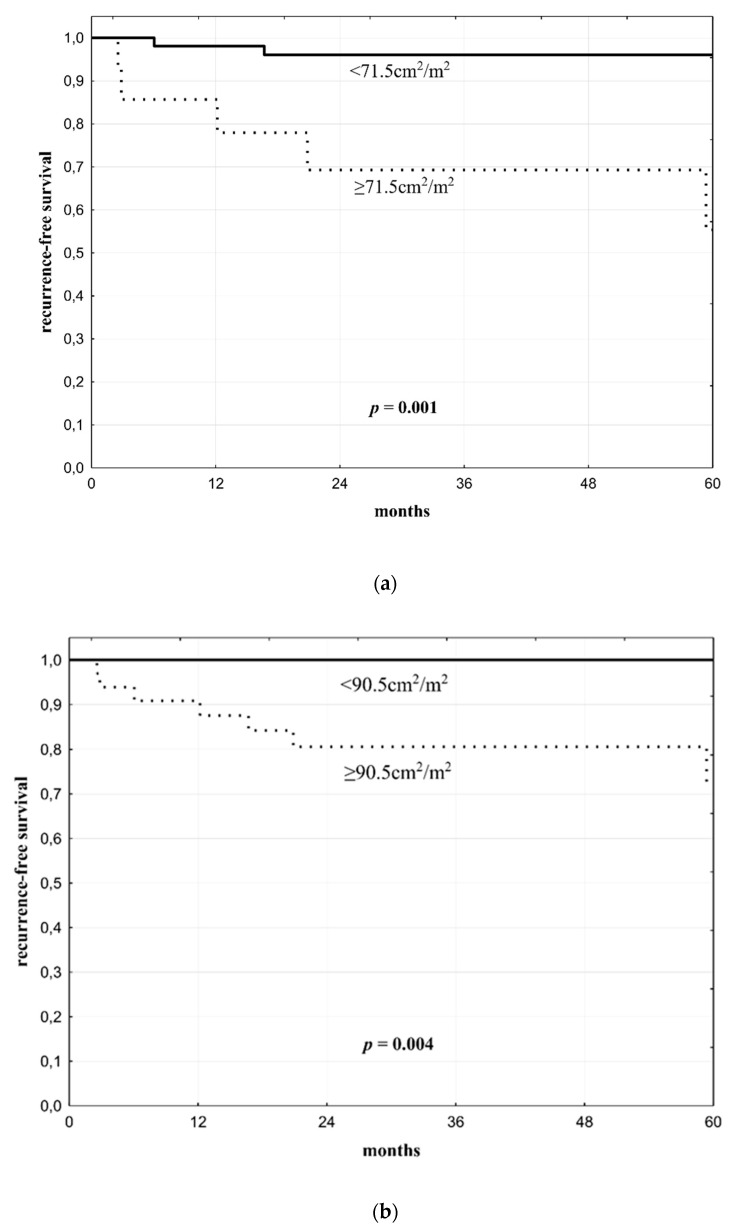
Recurrence-free survival after liver transplantation in HCC patients with (**a**) subcutaneous fat area <71.5cm^2^/m^2^ and ≥71.5cm^2^/m^2^ and (**b**) total fat area <90.5 cm^2^/m^2^ and ≥90.5 cm^2^/m^2.^ . (**a**) Patients with the amount of subcutaneous fat area above the cut-off value had lower recurrence-free survival rates (**b**) Patients with the amount of total fat area above the cut-off value had lower recurrence-free survival rates.

The optimal cut-offs for prediction of post-transplant HCC recurrence were 71.5 cm^2^/m^2^ for SFA and 90.5 cm^2^/m^2^ for TFA with AUCs of 0.772 and 0.767, respectively. Five-year recurrence-free survival rates were 96.0% in patients with SFA < 71.5 cm^2^/m^2^ as compared to 55.4% in patients with SFA ≥71.5 cm^2^/m^2^ (*p* = 0.001; Figure 3a). Five-year recurrence-free survival rates were 100% in patients with TFA <90.5 cm^2^/m^2^ as compared to 72.5% in patients with TFA ≥90.5 cm^2^/m^2^ (*p* = 0.004; Figure 3b).

In 33 patients with BMI within the range of 18.5–24.9 kg/m^2^, there were 3 (9.1%) with SFA ≥ 71.5 cm^2^/m^2^ and 7 (21.2%) with TFA ≥ 90.5 cm^2^/m^2^ (Table 4). On the contrary, out of 35 overweight patients (BMI category 25–29.9), there were 25 (71.4%) and 14 (40.0%) patients with SFA < 71.5 cm^2^/m^2^ and TFA < 90.5 cm^2^/m^2^, respectively. In nine obese patients (BMI ≥ 30 kg/m^2^), there was only one (11.1%) with SFA < 71.5 cm^2^/m^2^ and none with TFA < 90.5 cm^2^/m^2^. 

## 4. Discussion

Both obesity and sarcopenia are highly prevalent in a population of HCC patients with liver cirrhosis. Given the imperfect accuracy of current systems for evaluating patient eligibility, evaluation of the impact of body composition on post-transplant risk of recurrence seems clinically important. In contrast to studies performed in patients undergoing liver resection, no significant impact of sarcopenia on HCC recurrence risk was observed. Interestingly, the most important body composition factor influencing this risk was the quantity of subcutaneous adipose tissue. 

Patients with SFA exceeding 71.5 cm^2^/m^2^ were characterized by approximately 11-fold higher risk for post-transplant tumor recurrence than those with smaller amount of subcutaneous tissue. While a total amount of adipose tissue was also significantly associated with the risk of tumor recurrence, these seems to be attributable to the effects of subcutaneous fat given no significant effects of visceral fat area. This is clearly in contrast to previous studies, which results pointed towards the negative impact of visceral fat and irrelevance of subcutaneous tissue. In a recent study on 78 HCC liver transplant recipients increased visceral fat amount was significantly associated with post-transplant tumor recurrence, whereas no significant effects were found for the amount of subcutaneous adipose tissue [30]. Similarly, visceral to subcutaneous fat amount ratio was recently reported to increase the risk of HCC recurrence in patients undergoing liver resection [31]. In contrast, several studies identified high visceral fat amount to be a protective factor with respect to HCC recurrence after liver resection [21,32]. Nevertheless, given the high metabolic activity of visceral adipose tissue, it was expected to yield more effects on the risk of recurrence than the subcutaneous tissue [33]. The major negative impact of increased amount of subcutaneous adipose tissue is, therefore, a rather unexpected finding. Although this is the first study reporting the negative consequences of excessive subcutaneous tissue with respect to the risk of HCC recurrence, it was previously described as a risk factor for patients with prostate cancer [34,35]. 

While visceral adipose tissue is commonly considered to be metabolically more active than subcutaneous adipose tissue, recent findings point towards the important role in secretion of adipokines and potential influence on immune response of the latter. A recent study performed in middle-aged men indicated that expression of adipokines in subcutaneous adipose tissue is directly related to circulating adipokine profile, including leptin and adiponectin [36]. In a study on lung transplant recipients, subcutaneous but not visceral adipose tissue was associated with primary graft dysfunction [37]. In that study, pre-transplant leptin levels were highly correlated with subcutaneous adipose tissue index. Importantly, leptin was previously found to increase the metastatic potential of HCC cells through activation of several signaling pathways and to promote angiogenesis [38,39,40]. Further, leptin may promote creation of protumorigenic microenvironment by influencing cytokine profile and activity of matrix metalloproteinases [41]. In contrast, the actions of adiponectin, which levels decrease in obesity, include promotion of HCC cells apoptosis and anti-inflammatory effects [38,42]. Excessive adipose tissue is known to promote systemic pro-inflammatory response through activation of macrophages and secretion of multiple pro-inflammatory cytokines, including tumor necrosis factor α and interleukin 6, and therefore, promote post-transplant HCC recurrence [43]. Nevertheless, the mechanisms underlying the association between excessive subcutaneous adipose tissue and elevated risk of post-transplant HCC recurrence should be elucidated.

Sarcopenia was identified as a predictor of post-transplant HCC recurrence in a single study performed in recipients of living-donor liver transplantations [44]. The results of another study on recipients of living-donor liver transplantations pointed towards the negative impact of low skeletal muscle mass to visceral fat ratio, which is indicative of sarcopenic obesity [45]. Both studies were performed in Asian population, and similarly for the majority of those indicating the association between sarcopenia and HCC recurrence after liver resection [20,46,47,48,49,50]. The results of the present study indicate that sarcopenia does not increase the risk of HCC recurrence after deceased donor liver transplantation, at least in a European population. 

Notably, all of the body composition parameters analyzed on computed tomography images were significantly associated with patient BMI. Accordingly, increased BMI was associated with increased probability of excessive amount of both subcutaneous and visceral adipose tissue. Nevertheless, the results indicate that BMI itself is a highly imprecise parameter for evaluation of these two body composition components, as 9% and 21% of patients with normal weight had excessive amount of subcutaneous and visceral fat, respectively. Further, majority of overweight patients had low amount of subcutaneous adipose tissue and 40% of these patients did not have excessive amount of visceral fat. This is in line with the finding of previous studies [32]. Further, BMI was not found to be associated with outcomes of HCC patients after liver transplantation neither in present nor previous studies [51]. The necessity of evaluating subcutaneous and visceral adipose tissue on computed tomography in obese patients seems more controversial, as there was only one patient with SFA below the established limit in this population. However, these findings are limited by a relatively low number of patients with obesity in the present study and this, should be interpreted with caution, especially considering patient with excessive ascites. 

The method utilized in the present study for quantification of adipose tissue is similar to those described in other studies, except for a higher threshold for HU units (−150 HU versus −190 HU) [21,30,32]. However, as all the images were assessed individually, this was unlikely to bring remarkable differences. While psoas muscle thickness was previously assessed instead of psoas muscle area, the method used to evaluate muscular tissue in the present study was commonly utilized previously [44,46,47,48,49,50].

Neoadjuvant treatment was commonly utilized in the study performed in liver transplant recipients that pointed towards the significant prognostic role of visceral adipose tissue [30]. Conversely, patients in the present study did not undergo neoadjuvant treatment and due to selection bias, had generally lower tumor burden with less biologically aggressive tumors, as indicated by lower rate of microvascular invasion and poor differentiation. Therefore, the results need to be validated in a larger cohort of patients in order to evaluate potential differences in prognostic roles of visceral and subcutaneous adipose tissue depending on tumor burden and biological aggressiveness. Similarly, patients included in the study pointing towards the association between sarcopenia and tumor recurrence were all beyond the limits of Milan criteria and had more excessive tumor burden than those included in the present study [44]. Further, they were recipients of living-donor liver transplantation. Accordingly, the results of the present study are limited to more homogeneous group of patients at generally lower risk of post-transplant HCC recurrence.

The present study is subject to similar limitations. First, it was retrospective in nature and thus prone to all limitations of retrospective studies. Second, there was a low absolute number of the observed tumor recurrences, precluding performance of multivariable analyses. This also results in a higher risk of type II error for evaluating the effects of visceral fat amount and sarcopenia on the risk of HCC recurrence. On the other hand, this was related to selection of more homogeneous population of HCC patients for the present study, as all patients undergoing pre-transplant neoadjuvant treatment were excluded. Moreover, the number of patients included in the present study is similar to those included in other studies focused on liver transplantation [30,44].

## 5. Conclusions

In conclusion, the results of the present study indicate that excessive amount of subcutaneous adipose tissue is associated with increased risk of tumor recurrence and may be considered as another factor potentially useful in selection of HCC patients for liver transplantation. Neither visceral adipose tissue nor sarcopenia seem to be related with the risk of HCC recurrence following liver transplantation.

## Figures and Tables

**Table 1:** Baseline characteristics of the study group.

	Medians and IQRs or Numbers and %
Age (years)	56 (52–61)
Sex	
Male	59 (76.6%)
Female	18 (23.4%)
HCV	58 (75.3%)
BMI (kg/m^2^)	25.20 (23.3–28.3)
Serum AFP (ng/ml)	13.1 (5.0–112.8)
Adipose tissue (cm^2^/m^2^)	
subcutaneous	51.7 (37.3–72.3)
visceral	27.8 (20.3–46.6)
total	83.4 (58.7–110.9)
Muscular tissue (cm^2^/m^2^)	
psoas major	6.2 (5.1–7.3)
total	50.1 (43.1–56.4)
Time between LT and the last CT scan (days)	51 (26–95)
Tumor characteristic	
number of tumors	1 (1–2)
size of the largest tumor	25 (15–35)
microinvasion	14 (18.67%)
poor tumor differentiation	9 (11.69%)
fulfillment of Milan criteria	53 (68.83%)

IQRs—interquartile ranges, HCV—Hepatitis C Virus, BMI—Body Mass Index, AFP—alpha-fetoprotein, LT—Liver Transplantation, CT—Computed Tomography.

**Table 2:** Correlations between Body Mass Index and adipose and muscular tissue areas in hepatocellular carcinoma (HCC) patients undergoing liver transplantation.

	R	*p*
Subcutaneous Fat Area (SFA)		
whole group	0.735	*p* < 0.001
males	0.756	*p* < 0.001
females	0.803	*p* < 0.001
Visceral Fat Area (VFA)		
whole group	0.569	*p* < 0.001
males	0.509	*p* < 0.001
females	0.576	*p* = 0.012
Total Fat Area (TFA)		
whole group	0.710	*p* < 0.001
males	0.700	*p* < 0.001
females	0.803	*p* < 0.001
Psoas Muscle Area (PMA)		
whole group	0.453	*p* < 0.001
males	0.344	*p* = 0.008
females	0.606	*p* = 0.008
Total Skeletal Muscle Area (TSMA)		
whole group	0.529	*p* < 0.001
males	0.491	*p* < 0.001
females	0.667	*p* < 0.002

**Table 3:** Risk factors for tumor recurrence after liver transplantation for HCC.

Factor	HR	95% CI	*p*
Area of:			
subcutaneous fat ^a^	1.026	1.005–1.047	0.013
visceral fat ^a^	1.025	0.982–1.070	0.264
total fat tissue ^a^	1.026	1.005–1.049	0.017
psoas major ^a^	1.409	0.893–2.225	0.141
total skeletal muscle ^a^	1.024	0.934–1.122	0.618
Age ^b^	0.997	0.901–1.103	0.952
BMI ^c^	1.183	0.956–1.463	0.122
Tumor characteristics:			
number of tumors ^d^	1.254	0.983–1.599	0.068
size of the largest tumor ^e^	1.069	1.020–1.119	0.005
microvascular invasion	5.780	1.284–25.641	0.022
poor tumor differentiation	3.534	0.685–18.182	0.132
fulfillment of Milan criteria	100% vs. 53.3% *		*p* < 0.001 *
Donor age ^b^	0.999	0.938–1.063	0.965
MELD points ^f^	0.995	0.846–1.170	0.949
Serum AFP ^g^	1.428	1.106–1.843	0.006
Female sex	0.627	0.075–5.217	0.666
HCV	0.903	0.100–2.041	0.301

HR—Hazard Ratio; 95% CI—95% Confidence Interval; BMI—Body Mass Index; MELD—Model for End-stage Liver Disease; AFP—alpha-fetoprotein; HCV—Hepatitis C Virus; HRs were given per: a—1 cm^2^/m^2^ increase; b—1 year increase; c—1 kg/m^2^ increase; d—per 1 tumor increase; e—per 1 mm increase; f—1 point increase; g—1 log ng/ml increase; * due to no tumor recurrences in the group of patients fulfilling Milan criteria, Cox proportional regression could not be performed; the statistical significance was based on comparison of survival curves.

**Table 4:** Distribution of HCC patients undergoing liver transplantation with SFA and TFA under and above established thresholds according to BMI category.

Body Mass Index (BMI) (kg/m^2^)	Subcutaneous Fat Area (SFA)		Total Fat Area (TFA)	
	Value (cm^2^/m^2^)	Number of Patients	Value (cm^2^/m^2^)	Number of Patients
18.50–24.99	<71.5 ≥71.5	30 (90.9%) 3 (9.1%)	<90.5 ≥90.5	26 (78.8%) 7 (21.2%)
25.00–29.99	<71.5 ≥71.5	25 (71.4%) 10 (28.6%)	<90.5 ≥90.5	14 (40.0%) 21 (60.0%)
≥ 30.00	<71.5 ≥71.5	1 (11.1%) 8 (88.9%)	<90.5 ≥90.5	0 (0.0%) 9 (100.0%)
